# On the Ablation Behavior of Carbon Fiber-Reinforced Plastics during Laser Surface Treatment Using Pulsed Lasers

**DOI:** 10.3390/ma13245682

**Published:** 2020-12-12

**Authors:** Jana Gebauer, Maximilian Burkhardt, Volker Franke, Andrés Fabián Lasagni

**Affiliations:** 1 Business Unit Microtechnology, Fraunhofer Institute for Material and Beam Technology IWS, Winterbergstraße 28, 01277 Dresden, Germany; burkhama@freenet.de (M.B.); volker.franke@iws.fraunhofer.de (V.F.); 2 Institute of Manufacturing Technology, Faculty of Mechanical Engineering, Technische Universität Dresden, George-Bähr-Straße 3c, 01069 Dresden, Germany

**Keywords:** pulsed laser, ablation threshold, carbon fiber-reinforced plastic, selective matrix removal

## Abstract

This contribution discusses the ablation phenomena observed during laser treatment of carbon fiber-reinforced plastics (CFRPs) with pulsed lasers observed employing laser sources with wavelengths of 355 nm, 1064 nm and 10.6 µm and pulse durations from picoseconds (11 ps) to microseconds (14 µs) are analyzed and discussed. In particular, the threshold fluence of the matrix material epoxy (EP) and the damage threshold of CFRP were calculated. Moreover, two general surface pretreatment strategies are investigated, including selective matrix removal and structure generation through indentation (ablation of both, matrix material and fibers) with a cross-like morphology. The surfaces obtained after the laser treatment are characterized by means of optical and scanning electron microscopy (SEM) and Fourier transform infrared spectroscopy is employed for the analysis of composite and constituent materials epoxy and carbon fibers. As a result, different ablation mechanisms, including evaporation and delamination are observed, depending on the employed laser wavelength and pulse duration. For both 355 nm and 1064 nm wavelength, the laser radiation produces only partial ablation of the carbon fibers due to their higher absorption coefficient compared to the epoxy matrix. Although a selective matrix removal without residues is achieved using the pulsed CO_2_ laser. Differently, both constituent materials are ablated with the nanosecond pulsed UV laser, producing indentations. The sum of the investigations has shown that existing theories of laser technology, such as the ablation threshold according to Liu et al., can be applied to composite materials only to a limited extent. Furthermore, it has been found that the pronounced heterogeneity of CFRP mostly leads to an inhomogeneous ablation result, both when creating grooves and during selective matrix removal, where the carbon fibers influence the ablation result by their thermal conductivity, depending on fiber direction. Finally, despite the material inhomogeneity, a scanning strategy has been developed to compensate the heterogeneous ablation results regarding structure depth, width and heat affected zone.

## 1. Introduction

Currently, processes and products with a very low environmental impact are required in all aspects of human life, as in the automotive sector [1]. One key enabler for a better environmental friendliness is the employment of lightweight materials with reduced weight and increasing resource efficiency. Within these materials, fiber-reinforced plastic (FRP) offers an outstanding stiffness-to-weight-ratio, which means that they combine high mechanical performance with low weight [2]. However, FRPs show different challenges regarding production processes and applications compared to traditional common metal components. These comprehend inhomogeneous material structure (fibers and matrix), lower thermal resistance as well as low wear resistance [2]. Consequently, established manufacturing processes for metals need to be adapted or replaced by advanced surface treatments for tailored FRP modification. An approach is to employ chemical surface pretreatments to modify the materials surface, which, nevertheless, are considered disadvantageous in terms of process speed, complexity and environmental impact [3]. Furthermore, plasma treatments energetically activate the surface and generates free bonds on the polymer surface with a short half-life period [4]. Other well-known mechanical pretreatments, such as mechanical blasting or grinding, can be applied in order to roughen the surface [5,6] but they simultaneously can cause fiber damage and contamination of the soft plastic surface [7,8]. On the other hand, laser ablation has the potential to be a cost-effective solution to overcome those challenges, by selectively treating the FRP surface for adhesive joining [9,10,11,12] or injection molding processes [13,14,15]. In addition, laser structuring is a contact- and chemical-free process, which has already shown the capability to produce rough and enlarged joining surface [15,16,17]. Moreover, laser pretreatment is the only process that allows a localized pretreatment with micrometer accuracy without masking and thus offers significantly extended design freedom [8,13]. Latest investigations show promising results of laser structuring of FRP before applying a metal coating, which is necessary for local functionalization, such as wear-resistance [8]. In this framework, short and ultra-short pulsed lasers offer a clean surface pretreatment on fiber-reinforced plastics that can be utilized to reach the required surface roughness for mechanical bonding without thermally or mechanically damaging the composite material, especially the reinforcing fibers [8,18,19]. Pulsed laser processing has been applied on FRP for different purposes, ranging from surface cleaning [20,21], to roughening or selective matrix removal [8,10,11,12,16,18,21], hole drilling [22,23,24] and laser cutting [25,26], among others. Köckritz et al. [21] evaluated laser pretreatment as superior to conventional surface pretreatment methods in terms of the resistance to aging for adhesively bonded FRP.

Several research works have been also dedicated to analyze the interaction of laser radiation with carbon fiber-reinforced plastics (CFRPs). Romoli et al. [24] investigated the absorption behavior of different common polymer matrix materials including epoxy. The results show significant differences between the polymers in the spectral range of typical laser wavelengths between 355 and 1064 nm [24]. Consequently the interaction of laser radiation with matrix and composite material and the resulting ablation behavior are strongly depending on the material composition, meaning that material-specific ablation thresholds need to be determined. A common method for doing this is the D^2^-method according to Liu et al. [9,22]. Considering that FRP consists of two components, i.e., the plastic matrix and the fiber reinforcement, two different thresholds exist. In case of selective matrix removal, it is known that the threshold of the carbon fibers is higher than the threshold of the matrix material. Otherwise, the selective matrix removal would contain fiber damage [9,10,22]. In case of laser cutting of FRP parts, both components have to be ablated and consequently, the laser energy has to be set above the carbon fibers higher threshold.

Wolynski et al. and Oliveira et al. independently investigated the ablation threshold of CFRP. They reported that by applying accumulated laser energy on CFRP, firstly the epoxy matrix above the carbon fibers was removed, while higher energy densities were needed to ablate the under laying carbon fibers [9,22]. Wolynski et al. investigated the ablation thresholds of carbon fibers (embedded in epoxy matrix) for three wavelengths with a picosecond laser (7–9 ps pulse duration). The reported thresholds were 0.216 J/cm^2^ for 355 nm, 0.284 J/cm^2^ for 532 nm and 0.410 J/cm^2^ for 1064 nm wavelengths. The ablation threshold of the epoxy matrix is expected to be an order of magnitude lower compared to the carbon fibers and is not considered [22].

Using the same D^2^-method and additionally varying the scanning speed, the ablation thresholds of epoxy (0.33 J/cm^2^) and of the carbon fibers (0.52 J/cm^2^) were determined by Oliveira et al. with a femtosecond pulsed laser for a wavelength of 1024 nm [9]. The laser treatment was performed a few millimeters before the focal point (100 mm) resulting in a spot radius of 150 µm. The ablation thresholds of both components decrease with decreasing speed, caused by the accumulation of laser-induced defects in the material, which generally occurs when multiple laser pulses are applied (incubation effect). Using a wavelength of 1024 nm and 0.18 mJ of pulse energy, the selective removal of the epoxy resin could be achieved [9].

One characteristic of the laser treatment of CFRP is the variable dimension of the heat-affected zone (HAZ), which depends on the fiber direction [22]. Multiple material properties are causing this effect: on the one hand, the thermal conductivity of the carbon fibers is several times higher than that of the epoxy material (0.2 W/mK); on the other hand, the fibers also present a three times higher thermal conduction in longitudinal direction (4.9 W/mK for standard carbon fibers) compared to the transversal one (1.7 W/mK for standard carbon fibers) [2,22,27]. Moreover, the very high vaporization temperature of carbon fibers (3500 K) in contrast to the low decomposition temperature of the epoxy (770 K) leads to a degradation of the epoxy preferred in the longitudinal direction of carbon fibers [27]. Wolynski et al. observed a larger HAZ using 1064 nm wavelength compared to 532 nm, because epoxy matrix is optically transparent to the IR and shows higher absorption at 532 nm [22]. In order to overcome this inhomogeneity, Sato et al. introduced a differentiation of the HAZ in two parts, which are defined by the matrix evaporation zone (MEZ) and the resin alteration zone (RAZ). In the MEZ, the epoxy was evaporated by the laser radiation. In the RAZ, the resin was altered by the conducted heat around the kerf, which is expected to reduce the mechanical strength of the composite [28]. Negarestani [25] investigated the influence of defocusing on the HAZ, specifically the matrix recession and on the kerf width during laser cutting of CFRP under 9 bar gas pressure. The investigation was applied using an ytterbium doped fiber laser with 1070 nm wavelength. Varying the focal plane from −2.38 to 2.38 mm under oxygen, a slightly increased MEZ was detected at 2 mm, whereas the kerf width increased constantly [25].

Beside the ablation threshold and the fiber orientation, also a detailed understanding of ablation phenomena is required to achieve a defined laser ablation on CFRP. Akman and Kreling et al. described the ablation phenomena occurring during the laser treatment on CFRP. Akman et al. generated selective matrix removal on CFRP and compared a nanosecond pulsed laser with ultraviolet spectrum (355 nm) with a microsecond pulsed CO_2_ laser (10.6 µm wavelength). Since the UV laser provides a high photon energy (3.5 eV), a photochemical ablation process is induced, breaking the polymeric chemical bonds [10,26]. According to Kreling, this energy enables breaking a C-C-bond as cold ablation [11]. In contrast, the CO_2_ laser operates with lower photon energy (0.12 eV) and the laser beam interacts with the highly thermally conductive carbon fibers and indirectly heats up the epoxy matrix. This ablation process is described as a purely photothermal ablation mechanism [10,29]. Consequently, thermal degradation of the polymer occurs, weakening the interface between fibers and matrix [10]. Additionally, in the investigations of Akman et al., the nanosecond pulsed UV laser and the microsecond pulsed CO_2_ laser enable a selective matrix removal. The UV laser operated without using a focusing lens (approximately 5 mm^2^ spot area on the material surface) in contrast to the CO_2_ laser, operating at the focal point (spot diameter 200 µm). Differences in ablation results for both lasers were observed due to different ablation mechanism. While no residues remained when using the UV laser, the CO_2_ laser left ablation products in the shape of redeposited polymer [10].

All previous investigations were performed on CFRP, a compound material of plastic matrix and reinforcing carbon fibers. Consequently, the interaction of the laser beam with the matrix material also involves interactions of the laser beam with the carbon fibers, which are positioned underneath of the epoxy resin. Although this approach is reasonable for the common laser treatment, a differentiation of the interaction of the laser beam with the single materials of the composite material is required, since the ablation mechanism needs to be understood in detail. This would allow for a transfer of this process knowledge to CFRPs with deviating fiber volume content or other fiber orientation, such as woven fabrics. In addition, it is important to understand the development of accompanying symptoms, such as an irregular HAZ.

In this study, we investigate the interaction of various laser sources with the bare material components of CFRP, carbon fibers and epoxy resin. Gaining knowledge about the interaction of the laser beam with the plain epoxy on the one side and with the pure carbon fiber filaments on the other side will help to understand which effects lead to the final ablation results during the treatment on the composite material. Moreover, investigations on the optical properties and the ablation thresholds have been implemented both for the single materials (epoxy resin and carbon fibers) and for the composite (CFRP). Finally, two kinds of laser processes including the fabrication of indentations [8,30] and selective matrix removal were produced in the CFRP. In contrast to previous investigations, all experiments, from thresholds to laser structuring, were performed working on the focal point position. The thresholds are determined using single pulses instead of accumulated laser spots, preventing heat accumulation effects. Besides to the influence of the wavelength on the ablation mechanism also the influence of pulse duration is considered in this research. The influence of the carbon fiber on the ablation result in CFRP is discussed.

## 2. Materials and Methods 

The carbon fiber-reinforced epoxy composite (SGL Carbon, Wiesbaden, Germany) used in this work consisted of a thermoset matrix with unidirectional oriented carbon fibers with a degradation temperature of 543 K. For the matrix sample epoxy resin L plus hardener CL from R&G (Waldenbuch, Germany) was used. The produced epoxy plate is similarly highly transparent as the epoxy E501 in the CFRP. High tenacity carbon fibers ZOLTEK from Toray Group were used. These carbon fibers are considered as standard carbon fibers [2]. The CFRP and the epoxy plates were 2 mm thick, the carbon fibers were stacked up to 1.5–2.0 mm. The properties of the materials used are summarized in Table 1.

For the laser treatment, three different laser sources were used, from short pulse (SP) to ultra-short pulse (USP) and with wavelengths of 355 nm (UV), 1064 nm (IR-A) and 10.6 µm (IR-C). 

**Figure 1:**
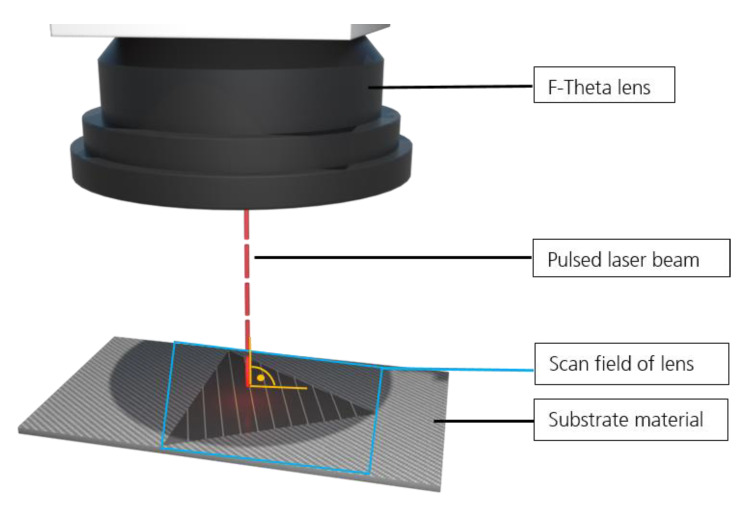
Laser setup showing the substrate positioned perpendicular to the incoming laser beam.

In Figure 1, the laser setup is depicted, showing that the substrate was positioned perpendicular to the lens of the laser system and was not moved during the laser process. All laser treatments were performed at the focal point of the corresponding lens, validated by stepwise movement of the lens (*z*-axis) until a circular focal spot appeared in the substrate.

The technical specifications and the optical set-ups are shown in Table 2.

For each of the three materials, the ablation threshold according to Liu et al. [31] was investigated. The Liu model requires a Gaussian distribution of the laser spot. Single pulses were applied on the epoxy, the carbon fiber filaments and the composite material. The sizes of the ablation areas were measured, rising with increasing pulse energy. Corresponding values of the squared diameter (D^2^) as derived from the experiments are drawn as a function of the applied fluence. The threshold was determined by the fluence value at which the beam diameter becomes zero (D^2^ = 0) [31,32]. For all pulse energies single pulses were applied 10 and 30 times for the epoxy and CFRP materials, respectively. The large number of measurements was chosen to compensate the variation of the measured sizes of the interaction area on the composite material.

Afterwards, the interaction of the laser beam with the CFRP was investigated for two kinds of laser structures. The selective matrix removal exposed the carbon fibers from the matrix material. Only the transparent epoxy was ablated. By generating indentations, concretely cross-like structures, either epoxy and carbon fibers were ablated. The grooves were produced perpendicular (90°) and parallel (0°) respectively to the fiber direction in the CFRP and rotated by ±45°.

The optical properties of the compound and the single materials (epoxy and carbon fibers) were investigated using different spectroscopy techniques. The UV-VIS-NIR-spectroscopy measurements were performed with a Cary 5000 (from Varian, Lake Forest, CA, USA) while for the Fourier transform infrared (FTIR) measurements, a spectroscope with Frontier (from Perkin Elmer, Waltham, MA, USA) was used. The fraction of the transmitted and reflected light permitted to calculate the absorbed radiation for each material depending on the wavelength. The measurements were conducted in the wavelength range from 0.3 to 15.0 μm using an integrating sphere. The samples were positioned both parallel and perpendicular to fiber direction in the spectrometer.

The surface of the laser treated surfaces was investigated with a light microscope (VHX-5000 from Keyence, Osaka, Japan), confocal microscopy (DCM3D from Leica, Wetzlar, Germany) and scanning electron microscopy (SEM, JSM-6610LV from Jeol, Freising, Germany). The SEM operated at 15 kV acceleration voltage and a working distance of 20 mm.

## 3. Results and Discussion

### 3.1. FTIR Spectroscopy

**Figure 2:**
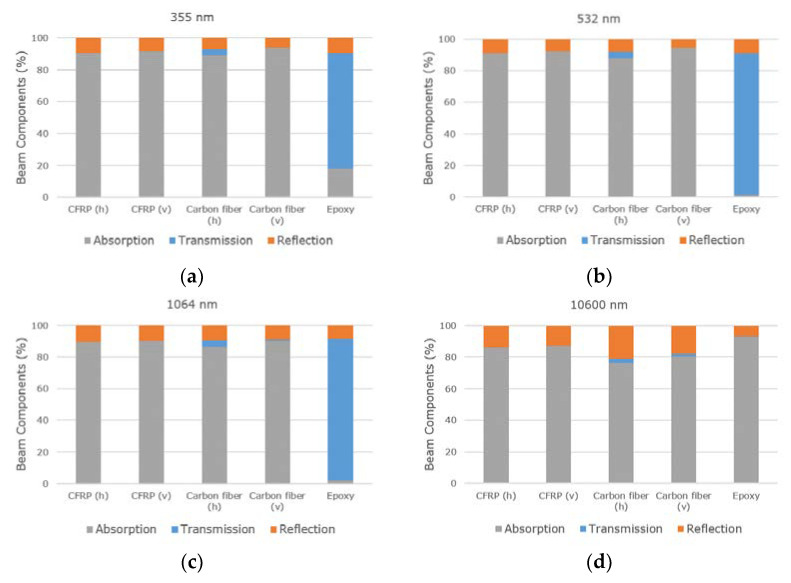
Percentage of absorbance, transmittance and reflectance for CFRP and carbon fibers horizontal (h) and vertical (v) to fiber direction and epoxy for the wavelengths (**a**) 355 nm; (**b**) 532 nm; (**c**) 1064 nm and (**d**) 10.6 µm.

The FTIR spectroscopy investigations of the composite and the individual materials provide information on their wavelength dependent absorption for the given thickness. Figure 2 shows the absorbance, transmittance and reflectance for the different materials (CFRP, carbon fiber and epoxy matrix) at the laser wavelengths of 355 nm, 532 nm, 1064 nm and 10.6 µm. From the Figure 2a–d, it is noticeable that the optical properties of the CFRP were similar to the carbon fibers for all wavelengths, showing an absorbance above 73%. In contrast, the epoxy material shows high transmittance (>70%) for 355–1064 nm and very low absorbance values, from 18% for 355 nm to almost 0% for 532 nm and 1064 nm wavelengths. The highest absorbance for the epoxy matrix was measured at 10.6 µm with 90%.

For the CFRP and the carbon fibers, the values for reflectance, transmittance and absorbance do generally not depend on the orientation (horizontal or vertical) of the fibers. The transmission part is slightly higher for horizontal orientated carbon fibers. Additionally, the absorption of CFRP was similar for all wavelengths, ranging from 86 to 91%, and the transmission was in all cases 0%. This means that the generation of indentations in CFRP might be possible with all wavelengths. 

Concerning the carbon fibers, at 355 nm wavelength, the vertical carbon fibers show the highest absorption with 94% and a transmission value of 0%. The composite material shows similar values with more than 90% absorption. The epoxy mostly transmitted 73% of the laser radiation and only 18% was absorbed.

For the 532 nm wavelength, the absorption of vertical carbon fibers was higher than 90%. A similar absorption value was observed for the CFRP. The epoxy material showed 90% transmission and 9% reflection. In contrast to the results of Wolynski et al. [22], no higher absorption in the CFRP was detected for 532 nm compared to 1064 nm, although the CFRP plates had a similar thickness of 1.9 mm (2.0 mm in this study). Wolynski et al. made this assumption based on the determined thresholds for both wavelengths using an ultra-short pulsed laser with pulse duration of 9 ps [22]. The absorption values of CFRP were not measured by spectroscopy. Due to the much higher radiation intensities during ultra-short pulsed laser processing, compared to a non-destructive spectroscopy measurement, non-linear absorption will occur such as multi-photon-absorption [33].

In contrast to the above described wavelengths, at 10.6 µm the highest absorption was measured in the epoxy resin with 93%, and the reflectivity was 7%. The carbon fibers absorbed the radiation up to 80%. The difference of 13% might offer a process window for selectively treating the epoxy. All in all, the wavelength of 10.6 µm is the only one offering a higher absorption in the epoxy material than in the composite or the carbon fibers.

Due to the very similar results obtained for both 532 nm and 1064 nm wavelengths, the wavelength of 532 nm was discarded for the rest of this research work.

### 3.2. Preliminary Considerations of the Ablation on the Composite Material

A mixture of two components in a composite material results in different local optical properties. Thus, the ablation behavior under laser treatment is influenced by the properties of both materials. Besides the optical properties, also the laser parameters, such as pulse duration and pulse energy, influence the laser-matter interaction. These are important parameters, which define the ablation result regarding the occurring ablation processes, the HAZ and the topography of the ablated region.

**Figure 3:**
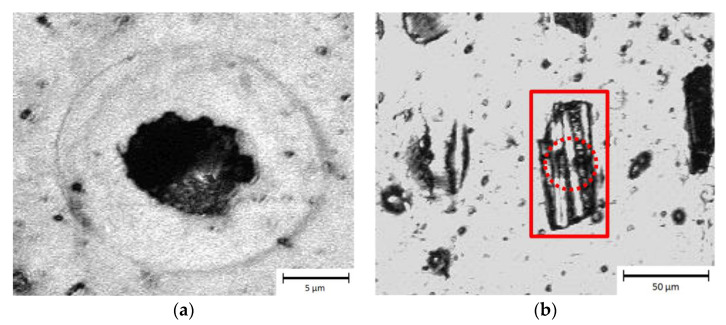
Microscope images of ablation area in bare epoxy (**a**) and carbon fiber-reinforced plastic (CFRP; **b**) using an SP-UV laser with a fluence of 19.1 J/cm^2^. The rectangle denotes the ablation area in CFRP after a single spot, while the dashed circle highlights the area where the carbon fibers were damaged.

The application of single laser pulses in the bare epoxy produced a homogeneous circular ablation area following the Gaussian distribution of the laser beam, as shown in Figure 3a. This area is surrounded by a concentric shadow with a diameter of 20 µm. This shadow could be traced back to dynamic processes producing laser supported absorption (LSA waves). In pulsed laser processing, the intensities are high enough so that the vaporization process starts very quickly under increasing pressure. The vapor or plasma, flowing off the surface of the interaction zone, transfers its impulse into the work piece and the immediately adjacent ambient gas is displaced. Consequently, gas-dynamic shock waves are formed, which propagate three-dimensionally from the interaction zone into the surrounding atmosphere [34]. Another reason for the shadow can be the creation of a HAZ or residues.

In contrast, the obtained ablation area in the CFRP (Figure 3b) presented a non-symmetrical shape, with a size of 70 µm in the longitudinal direction compared to the transversal direction with 30 µm (similar to diameter of the focused spot). An explanation of this behavior can be attributed to the thermal conductivity of carbon fibers, which is 4.9 W/mK along the fibers and only 1.7 W/mK in the perpendicular direction [1], leading to the heating of the material following mainly the fiber direction.

The size of the ablated region in the direction perpendicular to the fibers was very similar to the focal diameter (see dashed circle in Figure 3b), which means that the produced thermal stresses followed the heat flow direction. In consequence, the matrix material delaminated from the fibers. In addition, Chen et al. already reported that the heating of the carbon fibers could lead to a thermal degradation of the fiber-matrix-bonding [27]. Due to the weakened interface an enlarged area of epoxy resin can be removed easily. Consequently, beside the vaporization of the epoxy also the induced damages at the interface between both materials can explain the observed ablation results.

**Figure 4:**
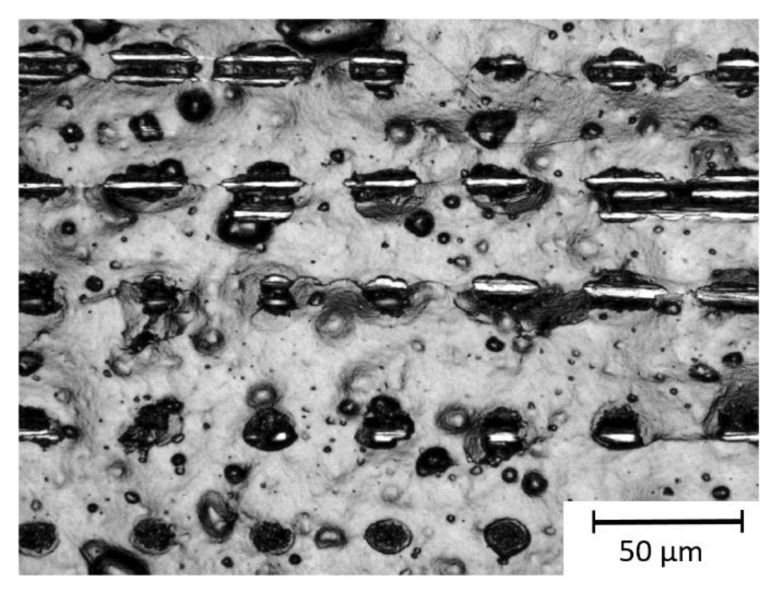
Microscope image of the ablation area in CFRP using the ultra-short pulse (USP) laser at a fluence of 4 J/cm^2^.

Figure 4 shows that the size of the ablated area in the CFRP by a single laser spot depends on the position of the carbon fibers. In particular, ablation primarily occurs at the fiber positions. Single laser pulses on CFRP left a visible interacting area, in case carbon fibers were present near the surface (first and second row in Figure 4). Otherwise, with the same laser energy a significantly smaller interaction area could be observed when a thicker epoxy layer covered the fibers (last row in Figure 4). Thus, the size of the ablation area also depends on the distance of the reinforcing fibers to the CFRP surface. In summary, the ablation of the epoxy material in the composite is strongly affected by the heating of the carbon fibers.

### 3.3. Calculation of Ablation Thresholds

**Figure 5:**
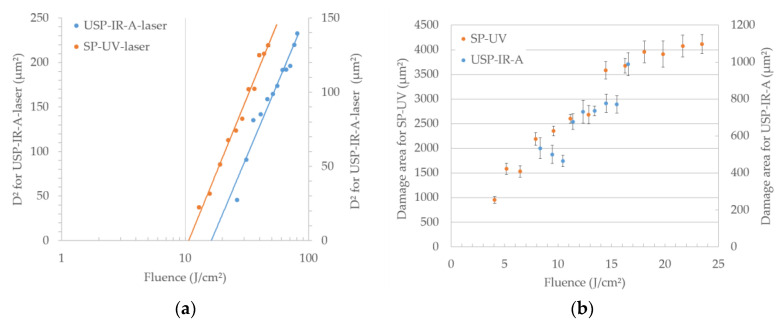
Ablation threshold of epoxy (**a**) and damage threshold of CFRP (**b**) versus fluence for the SP-UV laser and USP-IR-A laser.

It is well know that the threshold fluence values are material-specific for defined wavelength and pulse duration [35]. The ablation thresholds of pure epoxy for the SP-UV and USP-IR-A lasers were determined according to the model proposed by Liu et al. [31], by measuring the diameter (D) of the ablated area as a function of the laser fluence, as plotted in Figure 5a. In the case of the fiber material, a reliable measurement of the ablation area was not possible, due to the fact that the fibers absorbing the laser radiation are first cut and warp due to their internal stresses. This effect has been already observed by other authors, who outlined that there is any validated method to calculate the ablation threshold of carbon fiber filaments [15], since any influence of the surrounding matrix material has to be excluded.

For the CFRP material, due to the irregular shape of the ablated areas, it was not possible to determine the diameter D of the ablated area (see Figure 3b). In this case, the damage threshold [36,37,38] was calculated (instead of the ablation threshold) by measuring the total area of the affected region. The damaged area in CFRP is significantly enlarged in the longitudinal direction. Furthermore, it shows a large standard deviation. Considering this, no circular geometry can be assumed. Instead, a rectangular shape is assumed for the affected area. The damage thresholds were determined for the SP-UV and USP-IR-A lasers, as shown in Figure 5b. In case of the used CO_2_ laser (SP-IR-C), the ablation threshold could not be determined since the pulse duration of the laser source changed as a function of the laser power.

Using the USP-IR-A laser for the ablation of the epoxy material required a 50% higher fluence of 16.3 J/cm^2^ (24.5 µJ pulse energy) than the SP-UV laser with 10.7 J/cm^2^ (36.4 µJ pulse energy). This effect is mainly caused by the significantly lower absorption for 1064 nm (approximately 12 times) and the higher photon energy of the SP-UV laser. In this case, the standard deviation of the measured values for determining the ablation threshold in the epoxy was less than one square micrometer. Consequently, the reproducibility for the ablation threshold in epoxy is high.

The damage areas generated in the CFRP included both ablated epoxy and damage or partial ablation of the carbon fibers. Fluences above 8.3 J/cm^2^ (or 15.5 µJ pulse energy) were necessary to achieve ablation in CFRP with the USP-IR-A laser, while the SP-UV laser generated material ablation from 4.1 J/cm^2^ (or 24.0 µJ pulse energy). As previously mentioned, due to the irregular geometry of the ablated area in the CFRP, the ablation threshold could not be determined. Instead, the lowest pulse energy achieving material ablation in CFRP has been defined as a damage threshold. The damage areas show a high variation for both laser systems. The standard deviation of the damage areas was between 4% and 11% (26.6–62.7 µm^2^) for the USP-IR-A laser. Using the SP-UV laser, the standard deviation was 4–7% (69.3–265.5 µm^2^). The reproducibility of damage area was low despite the high number of measuring points. The high standard deviation as due to the inhomogeneity of the material and seemed to be the reason why for fluences below 11 J/cm^2^ there was not an increase of the damage area with increasing fluence using the USP-IR-A laser shown, as expected. The measure points concerned are within their standard deviation around 50 µm^2^. Due to the influence of the position of the reinforcing fibers (explained in Section 3.2), the damage threshold is just an indicative value for how much fluence is required for a material removal in the CFRP. The determined thresholds for epoxy and CFRP are summarized in Table 3. 

The damage threshold of the CFRP approximately corresponds to the half of the ablation threshold of the pure epoxy plate. The embedded carbon fibers lower the threshold significantly. This shows that the carbon fibers promote laser ablation in the CFRP materials.

### 3.4. Laser Based Fabrication of Textured Surfaces in CFRP

Based on the findings from the previous sections, two different laser treatments were selected for the fabrication of textured surfaces on the CFRP material: (1) fabrication of cross-like textures based on indentations and (2) the selective matrix removal. Table 4 shows the used laser parameters for both cases.

The cross-like structures were produced using fluence values from 8 to 17 J/cm^2^. For the SP-UV and USP-IR-A lasers, the used laser fluence was at least two times the damage threshold of the composite to ensure a homogeneous ablation process and to achieve an efficient ablation rate.

#### 3.4.1. Indentations with Cross-Like Structure Geometry

**Figure 6:**
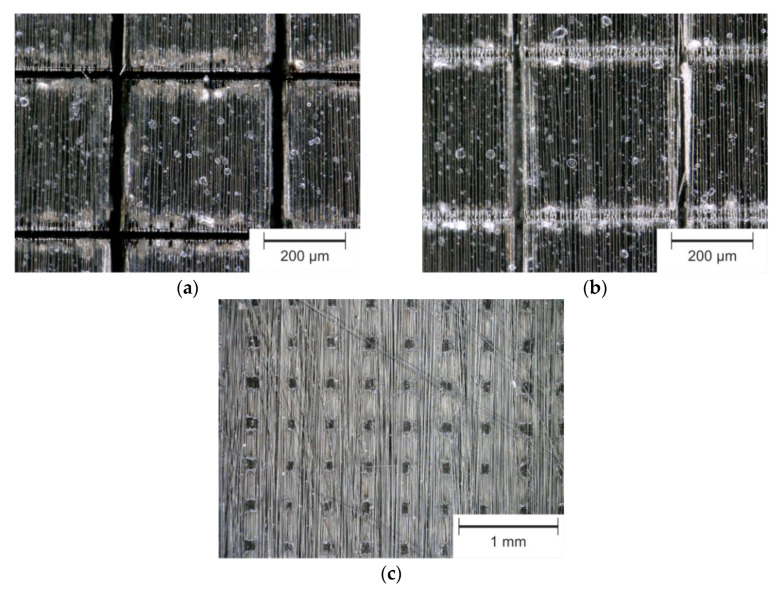
Optical microscope images of fabricated cross-like structures with 400 µm line distance using SP-UV (**a**), USP-IR-A (**b**) and SP-IR-C (**c**) laser sources.

Results of generated cross-like structures with the three selected laser systems (see Table 4) are shown in Figure 6.

The fabrication of narrow cross-like structures was successful with the laser systems SP-UV and USP-IR-A, as shown in Figure 6a,b. In contrast, using the SP-IR-C laser the carbon fibers got exposed along the interaction area with the laser beam (Figure 6c), exposed by degradation of the epoxy. Between these lines, the matrix material remained unaffected. High power distributed over long pulse duration of 8 µs prevented ablation of the carbon fibers. Increasing the laser power and/or decreasing the scanning speed caused thermal damage to the composite, due to flame formation in the processing area. Finally, no cross-like structures could be achieved using the SP-IR-C laser, since thermal damage of the surrounding composite could not be avoided, in order to maintain the mechanical strength of the component. In Figure 6b, it can be seen that the lines fabricated in fiber direction are deeper compared to the lines produced perpendicularly.

**Figure 7:**
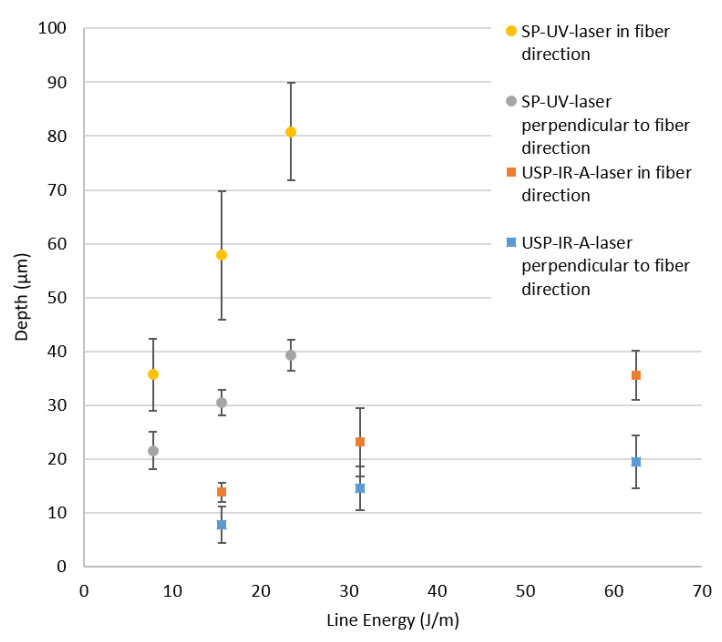
Depth of indentations in CFRP as function of the line energy depending on fiber direction for SP-UV and USP-IR-A laser sources. The dashed lines are only to guide the eyes.

Figure 7 shows the depths of the indentations as a function of the line energy in relation to the fiber direction. The line energy *E_line_* describes the amount of applied energy along one groove. It is determined using Equation (1):

*E_line_* = *P_av_*·*n*/*v* (1)

where *P_av_* is the average laser power, *v* is the scanning speed and *n* is the number of repetitions. The Formula (1) refers to the line energy according to Li et al. [39] for one pass, extended by the number of repetitions.

Using the SP-UV laser source with a line energy of 20 J/m, the produced indentations were 80.8 µm and 39.3 µm deep in the parallel and perpendicular directions, respectively, that is the groove depth in the fiber direction was about twice the groove depth in the perpendicular direction. The significantly higher thermal conductivity of the carbon fiber in the fiber direction and the identically oriented scan direction results in heat accumulation. This leads to an increased ablation rate in the fiber direction. In addition, when individual fibers are removed from the matrix, they can completely detach from the groove (only possible in fiber direction).

Additionally, from Figure 7, it can be seen that the USP-IR-A laser produced significantly less deep lines compared to the SP-UV laser. Considering that the absorption value is the same for 355 nm and 1064 nm (90%), the significantly lower pulse duration of the USP-IR-A laser system represents the main difference (30 ns vs. 0.01 ns). This led to a significantly lower peak power and thus resulting in a lower ablation rate.

**Figure 8:**
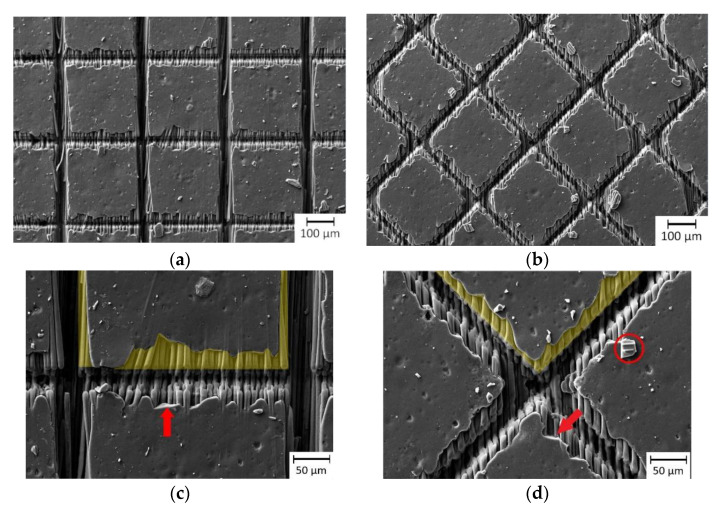
Generated cross-like structures by SP-UV laser 0°/90° (**a**,**c**) and ±45° to fiber direction (**b**,**d**). The red arrows denote sharp edges in the MEZ and the red circle an epoxy particle, chipped off. The yellow marked areas show the MEZ exemplarily for one square.

By individually adapting the number of repetitions for each scanning direction, homogeneous depths might be achieved for grooves parallel and perpendicular to the fiber orientation. However, also the scanning direction related to the fiber direction can be changed, as shown in Figure 8a,b. Comparing Figure 8a,b, it is noticeable that a more uniform ablation result was obtained due to the changed scanning direction.

**Figure 9:**
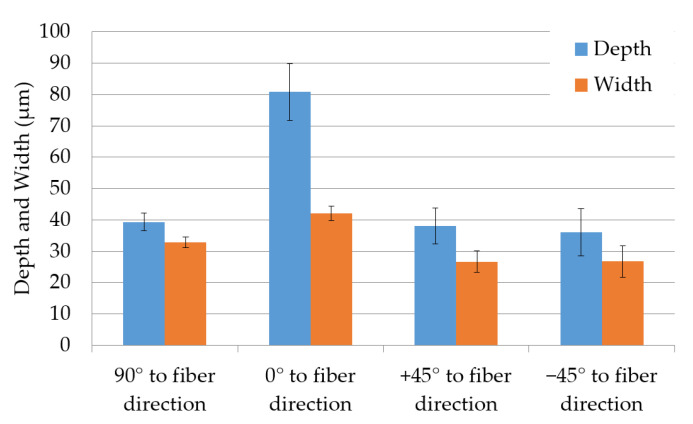
Depth and width of indentations dependent on fiber direction for a repetition number of six with SP-UV laser.

Figure 9 illustrates the influence of fiber direction to the resulting groove depth and width. As it can be seen in the Figure 9, for the directions of 90°, +45° and −45°, being 90° perpendicular to the fibers, both depth and with of the indentations were very similar. Additionally, the grooves in the fiber direction show the doubled depth compared to the perpendicular direction, which was explained previously. Clearly, the best results are obtained by cutting the fibers with the same angle (scanning directions of +45° and −45°), since the heat conduction became identical for both scanning directions. In the last case, the depth and width of the indentations were 37 µm and 27 µm, respectively.

The influence of the dependency of the fiber orientation can also be appreciated with more detail by analyzing the matrix evaporation zone (MEZ), which has different characteristics depending on the orientation of the fibers (Figure 8c,d). The MEZ is characterized by the fact that the matrix material was partly ablated. This is due to the heat conduction from the laser interaction area (in the groove) via the carbon fibers into the interior of the squares. For a cross-like structure orientated by ±45° to the fiber direction, the produced grooves show a more homogeneous MEZ (Figure 8d) compared to 0°/90° (Figure 8c). In the SEM images, also scattered sharp edges (marked by red arrow in Figure 8c,d) are visible. Fragments of the epoxy are present (red circle in Figure 8d). In contrast to the smooth edges, caused by the evaporation, the sharp edges indicate that the epoxy chipped off. All in all, the MEZ seems to have been influenced by a mixture of thermal effects. On one hand, by the evaporation of the epoxy resin and on the other hand by energy impulses that cause the epoxy resin to be chipped/splintered off.

#### 3.4.2. Selective Matrix Removal Texturing

**Figure 10:**
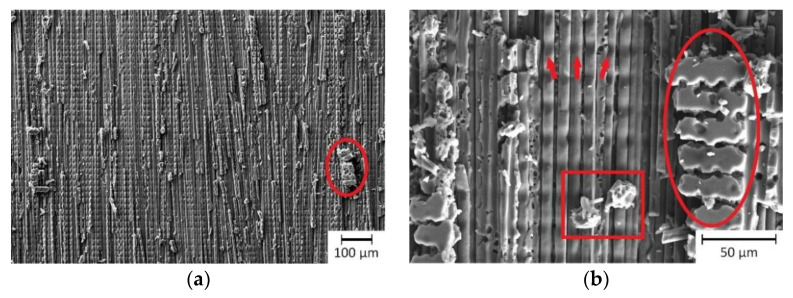

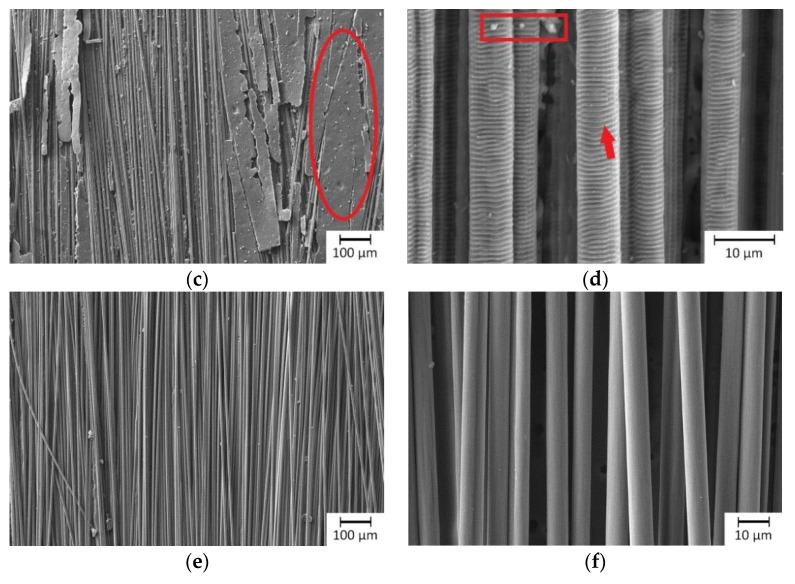
SEM images for investigation of exposing the fibers by selective matrix removal with SP-UV laser (**a**,**b**), USP-IR-A laser (**c**,**d**) and SP-IR-C laser (**e**,**f**). The red circles denotes the remaining epoxy resin, the red rectangle denote residues and the red arrows highlight damage of carbon fibers (in (**b**)) and LIPSS (in (**d**)).

Examples of the selective matrix removal strategy, performed with three different laser systems are shown in Figure 10a–f.

Using the SP-UV laser, neither material ablation nor ablation including fiber damage was achieved. Hence, different processing parameters were chosen, which provided a compromise between maximized fiber exposure and as less fiber damage as possible. The treatment with the SP-UV laser only exposed the upper surface of the carbon fiber filaments (Figure 10a,b). Partly, the matrix material still covered the fibers. In the remaining epoxy matrix, single ablation traces of the laser beam can be observed (highlighted by red ellipses). Furthermore, the SEM images indicate a slight ablation of the fibers resulting in narrow grooves across the fibers, marked by red arrows in Figure 10b. Considering the absorption value of 90% of the composite and the carbon fibers, in contrast to the low absorption of 20% of the epoxy material, it is possible to explain the partly remaining epoxy over the fibers. Clearly, for this wavelength the interaction of the laser beam with the carbon fiber predominated. The sharp edges of the remaining epoxy, all over the laser treated area, refer to an ablation process at least partly based on interfacial stresses (chipping). The traces in the remaining epoxy validate a photochemical ablation mechanism, as was previously explained by Akman et al. [10] and Kreling et al. [11] for the treatment of plastic-based materials with ultraviolet laser radiation. However, a thermal ablation mechanism, especially of the carbon fibers cannot be excluded. Contrary, according to the investigations of Akman et al., a residue-free selective matrix removal was possible, using a nanosecond pulsed UV laser without focusing [10].

Figure 10c,d show the CFRP surface after selective matrix removal by USP-IR-A laser. A cleaning step with compressed air was required for removing the detached matrix layer sticking on the exposed fibers. The carbon fibers at the surface were predominantly more affected than the epoxy material. Single areas show an insufficient ablation result by the remaining matrix material. The surface of these areas is very smooth and unaltered. There is no indicator for any interaction between the laser beam with the remaining epoxy. Since the transmittance at the wavelength 1064 nm was close to 90% in the epoxy and the absorption was negligible, the material ablation must be caused only by the interaction of the laser radiation with the carbon fibers. Furthermore, as it can be seen in Figure 10d, the exposed fibers became visible. Similar to the investigation of Oliveira et al., the carbon fibers show at their surface the so called laser-induced periodic surface structures (LIPSSs), which are typical for laser treatments with ultra-short pulses. Periodicity and orientation of the LIPSS depend on the wavelength, angle of incidence, laser beam polarization and fluence [9]. With a period of 800 nm, this LIPSS (highlighted by red arrow) can be classified as low-spatial frequency LIPSS, which are oriented perpendicular to polarization of the laser [9,40]. This is in agreement with the used polarization direction in our experiments (parallel to the carbon fiber direction). These topographical surface elements can for instance enhance bonding strength in adhesive joining by the enlarged surface area and by interlocking contribution. Due to the small size of the produced topographical features (submicrometer-range), compared to the diameter of the carbon fiber (10 µm) a weakening of the mechanical strength of the fibers is not expected [9]. In consequence, the presence of the LIPSS features confirms that the laser beam interacted mainly with the carbon fibers. Once the laser radiation was absorbed by the fibers, also interfacial stresses could contribute to the detachment of the matrix from the fibers. It has to be mentioned also, that for both laser treatments, residues of the epoxy matrix were still visible (see Figure 10b,d for SP-UV and USP-IR lasers, respectively).

The most homogeneous results were achieved using the SP-IR-C laser (Figure 10e,f). In this case, the matrix material was totally ablated giving rise to exposed fibers (Figure 10f). Neither structural damage nor residues could be observed on the fibers.

For carbon fiber-reinforced plastics, generally near the surface there is a heterogeneous distribution of the reinforcing fiber. In consequence, there are areas with a thicker layer of the matrix material while for the fiber located close to the surface, the amount of epoxy over the fibers is very thin [20]. These non-homogeneities led to different ablation results for both USP-IR-A and SP-UV lasers. Contrary, the comparatively long pulses of the CO_2_ laser and the high absorption (>90%) of epoxy material at the wavelength 10.6 µm led to a strong heating of the complete composite, and as mentioned above, the epoxy material could be successfully removed. Furthermore, several layers of fibers became completely exposed when using this wavelength. In contrast to Akman et al. [10], no redeposition of ablation products was found on the treated surface. The theory of Akman et al. that the vaporization of the epoxy occurred by thermal conductivity of the carbon fibers, is a concomitant effect leading to the degradation and removal of epoxy in deeper laying regions [10].

## 4. Conclusions

In this work, the ablation phenomena observed during the laser treatment of carbon fiber-reinforced plastics (CFRPs) with pulsed lasers operating at different wavelengths were investigated. Three wavelengths (namely 355 nm, 532 nm and 1064 nm) were employed and induced in proportionally similar absorption, transmission and reflection in the material. It was found that the carbon fiber or the composite material CFRP absorbed about 90% of the light, whereas the light in the epoxy was not absorbed, but transmitted to 90%. Only at 355 nm, the epoxy resin shows 20% absorption. At a wavelength of 10.6 µm, the absorption values for CFRP and carbon fiber were over 80% and the epoxy resin had an absorption value of 93%.

As far as the ablation mechanisms were concerned, it has been found that the thermal conductivity of the carbon fiber led to an asymmetric material removal in CFRP already at single laser spots, significantly increased in fiber direction. Moreover, the size of the ablation area in CFRP also depended on the distance between the reinforcing fibers and the CFRP surface: the more carbon fibers are located near the surface in the interaction area with the laser spot, the larger the material ablation. For the SP-UV laser and the USP-IR-A laser, the ablation thresholds for the epoxy were 10.7 J/cm^2^ and 16.3 J/cm^2^, respectively. Due to the asymmetrical ablation area in the CFRP the ablation threshold according to Liu et al. cannot be applied. Instead, damage thresholds for the composite material were determined, being (4.1 J/cm^2^ and 8.3 J/cm^2^) for 355 nm and 1064 nm wavelengths, respectively.

Furthermore, cross-like structures could be produced with both the SP-UV and the USP-IR-A lasers. In this case, the interaction of the laser beam with the carbon fibers was predominant. A pronounced dependence of the groove shape (depth and width) with the fiber direction was observed. Especially, the groove depth was significantly higher (about twice) when this is parallel to the fiber direction, due to higher thermal conductivity of the fibers compared to the matrix. Both the groove shape and the HAZ could be homogenized by rotation of the grooves from 0/90 to ±45°.

All three laser systems enabled a selective matrix removal texture of varying quality. The best results in terms of ablation quality could be achieved with the SP-IR-C laser (CO_2_), obtaining clean and free fibers without the presence of epoxy residues. In this case, a photothermal ablation mechanism was predominant.

## Figures and Tables

**Table 1:** Properties of materials.

Material	Name	Company	Thickness	Flexural Strength (MPa)	Tensile Strength (MPa)	Glass Transition Temperature T_G_ (K)	Density (g/cm³)
Epoxy Resin	Resin L + Hardener CL	R&G	2 mm	137	82.8	>363	1.15
Carbon Fiber	Zoltek^TM^ PX35	Toray Group	7.2 µm	-	4.1 ^1^	923	1.81
CFRP	Sigrapreg C U600-0/SD-E501/33%	Sgl carbon	2 mm	1400 ^1^	2000 ^1^	383 (Epoxy)	-

^1^ in longitudinal direction.

**Table 2:** Overview for used laser systems with their optical setups.

Description	SP-UV	USP-IR-A	SP-IR-C
Laser type	Nd:YAG	Nd:YAG	CO_2_
Wavelength (nm)	355	1064	10,600
Pulse duration (s)	30 × 10^−9^	10 × 10^−12^	3–400 × 10^−6^
Maximum power (W)	20	30	250
Focal length (mm)	160	80 ^1^/255 ^2^	200
Focal radius (µm)	13.4	7.8 ^1^/27.2 ^2^	213
Fluence range (J/cm^2^) in bare epoxy	4.1–23.5	8.3–38.0	-
Fluence range (J/cm^2^) in CFRP	2.7–23.5	1.3–38.0	2.4–7.9

^1^ for cross-like structure, ^2^ for selective matrix removal.

**Table 3:** Determined thresholds for CFRP and epoxy (EP).

Material	Ablation Threshold of EP (J/cm^2^)		Damage Threshold of CFRP (J/cm^2^)	
	SP-UV	USP-IR-A	SP-UV	USP-IR-A
EP	10.7 ± 0.6	16.3 ± 0.9	-	-
CFRP	-	-	4.1	8.3

**Table 4:** Laser parameters to generate indentations in form of a cross-like structure and to selectively remove the matrix on the CFRP.

Structure	Laser	Frequency (kHz)	Power (W)	Hatch Distance (µm)	Scanning Speed (mm/s)	Repetition Number (-)	Fluence (J/cm^2^)
Cross-like structure	SP-UV	130	7.14	400	800	2/4 ^1^/6 ^2^	9.41
	USP-IR-A	100	3.13	400	800	4/8/16	16.80
	SP-IR-C	30	33.00	400	800	3	7.93
Selective Matrix Removal	SP-UV	90	4.23	20	1200	2	6.38
	USP-IR-A	100	2.90	20	800	2	1.25
	SP-IR-C	10	33.90	88	2500	2	2.38

^1^ grooves 0° and 90° to fiber direction, ^2^ grooves +45° and −45° to fiber direction.
